# ﻿Taxonomic study of *Hydnoporia* (Hymenochaetales, Hymenochaetaceae) in East Asia with two new species

**DOI:** 10.3897/mycokeys.111.137347

**Published:** 2024-11-25

**Authors:** Minseo Cho, Yoonhee Cho, Sun Lul Kwon, Dohye Kim, Kentaro Hosaka, Young Woon Lim, Jae-Jin Kim

**Affiliations:** 1 Division of Environmental Science and Ecological Engineering, College of Life Sciences and Biotechnology, Korea University, 145 Anam-ro, Seongbuk-gu, Seoul, Republic of Korea; 2 School of Biological Sciences and Institute of Biodiversity, Seoul National University, Gwanak-ro, Gwanak-gu, Seoul, Republic of Korea; 3 BK21 FOUR R&E Center for Environmental Science and Ecological Engineering, Korea University, 145 Anam-ro, Seongbuk-gu, Seoul, Republic of Korea; 4 Department of Botany, National Museum of Nature and Science, 4-1-1 Amakubo, Tsukuba, Ibaraki 305-0005, Japan

**Keywords:** *Hymenochaete*, *Hymenochaetopsis*, novel species, phylogeny, *Pseudochaete*

## Abstract

The genus *Hydnoporia* (Hymenochaetales, Hymenochaetaceae) was first described by Murrill in 1907. However, species of *Hydnoporia* were subsequently reclassified into the genera *Hymenochaete*, *Hymenochaetopsis*, and *Pseudochaete* due to overlapping morphological characteristics, leading to multiple synonyms and confusion among researchers. Recent phylogenetic analyses based on multimarker datasets have clarified the diversity and relationships within *Hydnoporia*, but East Asian species remain underrepresented due to limited morphological data. To address this gap, we conducted a comprehensive morphological and phylogenetic analysis of East Asian *Hydnoporia* specimens using internal transcribed spacer (ITS) and translation elongation factor 1–α (*tef1*) regions. From 42 specimens, we identified six species, including two novel species, *Hydnoporiaorienticorrugata***sp. nov.** and *Hydnoporiasubtabacina***sp. nov.**, and we report *Hydnoporiarimosa* for the first time in Korea. Phylogenetic analyses also support the recombination of *Hymenochaeteintricata* and *Hymenochaetopsisrigidula* as *Hydnoporiaintricata***comb. nov.** and *Hydnoporiarigidula***comb. nov.**, respectively. By elucidating the phylogenetic relationships and morphological traits of *Hydnoporia* species from East Asia, this study contributes to a deeper understanding of the global diversity and phylogeny of the genus.

## ﻿Introduction

The genus *Hydnoporia* Murill. in the family Hymenochaetaceae (Hymenochaetales, Basidiomycota) was first described in 1907 and typified through *Hyd.olivacea* (Schwein.) Teixeira (≡*Sistotremafuscescens*). *Hydnoporia* is characterized by brownish basidiomes of diverse shapes, ranging from effused-reflexed to pileate, cylindrical to allantoid basidiospores, and setae of various sizes (Miettinen et al. 2019). *Hydnoporia* species are found across Asia, Europe, and North America (He and Li 2013; Miettinen et al. 2019; Palla et al. 2023). They inhabit the branches or stumps of conifer and deciduous trees and are classified as wood-decaying corticioid fungi (Miettinen et al. 2019). Until the 1990s, many corticioid species had been delimited based on their morphological traits (Parmasto 1995; Léger 1998). Due to their overlapping morphological characteristics, *Hydnoporia* species were previously placed under the genus *Hymenochaete* Lev., which includes species characterized by brown-colored basidiomes, small cylindrical basidiospores, and the presence of setae (Parmasto et al. 2014; Miettinen et al. 2019). The lack of distinguishable characteristics between the two genera prevented a clear separation despite the efforts of some taxonomists (Parmasto 1995; Léger 1998).

Technological advancements made DNA-based analyses possible during the 1990s and onwards. This led to many taxonomic revisions, including the division of *Hymenochaete* into two separate clades: *Hymenochaetesensu stricto* and *Pseudochaete* T. Wagner & M. Fisch (Wagner and Fischer 2002). However, the genus *Pseudochaete* was present under the botanical code in the green algae *Pseudochaete* W. West & G. S. West 1903, which was considered repetitious for microorganisms despite differences in lineages. For this reason, a new name *Hymenochaetopsis* S. H. He & Jiao Yang was introduced (Yang et al. 2016). However, none of these studies considered *Hydnoporia*, which had priority. *Hymenochaetopsis* and *Pseudochaete* eventually became synonyms of *Hydnoporia* (Miettinen et al. 2019). Additionally, the clade of *Hymenochaetetabacina* (Sowerby) Lèv. and its neighboring species were encompassed within *Hydnoporia* as it formed a sister clade to the type species, *Hyd.olivacea* (Miettinen et al. 2019; Wu et al. 2022).

Currently, 14 species are accepted in *Hydnoporia*, and 20–27 more species candidates have been revealed through multimarker-based phylogenetic analysis (Miettinen et al. 2019). However, previous studies did not fully cover East Asian specimens owing to the lack of detailed morphological data, and this was problematic because East Asia is expected to be the most diverse region for *Hydnoporia* species (He and Li 2013; Miettinen et al. 2019; Palla et al. 2023). To fully understand the global diversity of *Hydnoporia*, a detailed morphological and phylogenetic analysis of East Asian specimens is essential. This study assesses *Hydnoporia* specimens from the Republic of Korea and Japan based on multigenetic marker phylogeny (ITS+*tef1*) and morphological characteristics, and it describes two new species, *Hyd.orienticorrugata* sp. nov. and *Hyd.subtabacina* sp. nov. It furthermore notes one unrecorded species from the Republic of Korea, *Hyd.rimosa*. Additionally, we propose synonymizing *Hymenochaeteintricata* and *Hymenochaetopsisrigidula* as *Hyd.intricata* and *Hyd.rigidula*, respectively, considering the phylogenetic relationship of these species with *Hydnoporia*.

## ﻿Materials and methods

### ﻿Specimen collection

We studied 43 specimens labeled as *Hydnoporia*, *Hymenochaete*, *Hymenochaetopsis*, and *Pseudochaete* deposited in the Korea University Culture Collection (**KUC**), Seoul National University Fungus Collection (**SFC**), and National Institute of Biological Resources (**NIBR**) in this study. Specimens were collected nationwide from 2012 to 2021 and stored with silica gel under dry conditions to prevent contamination. A *Hyd.yasudai* specimen from **TNS** (Mycological Herbarium of the Department of Botany, National Museum of Nature and Science, Tsukuba, Japan) was also included.

### ﻿Molecular identification

Genomic DNA was extracted from the dried specimens using AccuPrep® Genomic DNA Extraction Kit (Bioneer, Daejeon, Korea). Primer sets ITS1F/ITS4, ITS1F/ITS4B, ITS5/ITS4, or ITS5/LR3 were used to amplify the nuclear ribosomal internal transcribed spacer (ITS) region (White et al. 1990; Gardes and Bruns 1993). For the translation elongation factor 1–α (*tef1*) region, the primer set 983F/1567R was used (Rehner and Buckley 2005). PCR products were purified using the AccuPrep® PCR Purification Kit following the manufacturer’s instructions. DNA sequencing was performed by Cosmogenetech (Seoul, Korea) using the amplified products. All sequencing results were edited using the SeqMan Lasergene package version 7.0.0 (DNAStar Inc., Madison, WI). The newly generated sequences were deposited in GenBank (Sayers et al. 2024; Table 1).

**Table 1.** List of analyzed *Hydnoporia* specimens with GenBank accession numbers of ITS and *tef1* sequences.

Species name	Specimen voucher	Country	GenBank accession no.^a^		References
			ITS	*tef1*	
*Hydnoporiacorrugata*	Jon Klepsland 2011-7-24 (O F-247869)^T^	Norway	MK514613	MK552138	(Miettinen et al. 2019)
	KCG001	Ireland	JQ246338	–	(Grundy et al. 2012)
	A6_wood_6	Great Britain	JN230421	–	(Grundy et al. 2012)
	B1_wood_inner	Ireland	JN230422	–	(Grundy et al. 2012)
	A3_wood_2	Great Britain	JN230419	–	(Grundy et al. 2012)
*Hyd.diffissa*	Otto Miettinen 19463 (H 7008917)^T^	USA, North Carolina	MK514611	MK552136	(Miettinen et al. 2019)
	Otto Miettinen 17127.4 (H)	USA, New York	MK514598	–	(Miettinen et al. 2019)
*Hyd.gigasetosa*	He1461	China, Yunnan	KT828671	–	(Yang et al. 2016)
	He1442	China, Yunnan	KT828670	–	(Yang et al. 2016)
***Hyd.intricata* comb. nov.**	**KUC20121123-03**	**Korea**	**PP992254**	**PQ066850**	**This study**
	**KUC20210428-20**	**Korea**	**PP992255**	**PQ066851**	**This study**
	**KUC20211030-01**	**Korea**	**PP992256**	**PQ066852**	**This study**
	**SFC20120820-11**	**Korea**	**PP992257**	–	**This study**
	**SFC20140313-01**	**Korea**	**PP992258**	**PQ066853**	**This study**
	**SFC20160920-36**	**Korea**	**PP992259**	**PQ066854**	**This study**
	**SFC20170822-68**	**Korea**	**PP992260**	**PQ066855**	**This study**
	**SFC20170908-28**	**Korea**	**PP992261**	**PQ066856**	**This study**
	He1181	China	JQ279609	–	(He and Dai 2012)
	He412	China	JQ279608	–	(He and Dai 2012)
	He21064	China	KC505556	–	Unpublished
*Hyd.lamellata*	Cui7629	China	JQ279603	–	(He and Dai 2012)
	Dai10527	China	JQ279605	–	(He and Dai 2012)
*Hyd.laricicola*	Viacheslav Spirin 5400 (H)	Russia, Khabarovsk	MK514606	MK552132	(Miettinen et al. 2019)
	Dai13458^T^	China, Heilongjiang	KT828672	–	(Yang et al. 2016)
	Dai11046	China, Nei Mongol	JQ279616	–	(He and Dai 2012)
	Wu 1207-122	China, Jilin	KT828673	–	(Yang et al. 2016)
*Hyd.latesetosa*	He492	China, Hainan	JQ716404	–	(He and Li 2013)
	He502^T^	China, Hainan	NR_120093	–	(He and Li 2013)
*Hyd.olivacea*	Otto Miettinen & Kelo Käppi 16044 (H 7005770)^T^	USA, Massachusetts	MK514612	MK552137	(Miettinen et al. 2019)
	P1201B	Peru	EU977192	–	(Smith et al. 2008)
	CMH529	USA, Missouri	KF800618	–	(Rittenour et al. 2014)
	f2Fc06	USA, Texas	GU721341	–	(Noris et al. 2011)
	319	Antarctica	KC785573	–	(Connell and Staudigel 2013)
	CFMR:DLL2011-223	USA, Wisconsin	KJ140712	–	(Brazee et al. 2014)
	CBS:126040	USA, North Carolina	MH864055	–	(Vu et al. 2019)
*Hyd.rhododendri*	N. Gerhold 2005-6-3	Austria	MK514593	–	(Miettinen et al. 2019)
	Viacheslav Spirin 6476 (H)	Russia, Primorsky Krai	MK514599	MK552127	(Miettinen et al. 2019)
	Viacheslav Spirin 6450 (H)	Russia, Primorsky Krai	MK514603	–	(Miettinen et al. 2019)
***Hyd.rigidula* comb. nov.**	SFC20140314-10	Korea	KX792928	–	(Kim et al. 2016)
	SFC20140411-08	Korea	KX792929	–	(Kim et al. 2016)
	SFC20140411-20	Korea	KX792930	–	(Kim et al. 2016)
	SFC20140703-24	Korea	KX792931	–	(Kim et al. 2016)
	**SFC20140723-16**	**Korea**	KX792932	**PQ066857**	(Kim et al. 2016), **This study**
	**SFC20160713-06**	**Korea**	**PP992262**	**PQ066858**	**This study**
	**SFC20170324-10**	**Korea**	**PP992263**	**PQ066859**	**This study**
	He379	China	JQ279613	–	(He and Dai 2012)
	He343	China	JQ279612	–	(He and Dai 2012)
***Hyd.rimosa***	**KUC20121109-19**	**Korea**	**PP992264**	–	**This study**
	Viacheslav Spirin 5277 (H)	Russia, Khabarovsk	MK514592	MK552122	(Miettinen et al. 2019)
	Viacheslav Spirin 5678 (H)	Russia, Khabarovsk	MK514594	MK552123	(Miettinen et al. 2019)
	Viacheslav Spirin 6104 (H)	Russia, Khabarovsk	MK514595	MK552124	(Miettinen et al. 2019)
*Hyd.subrigidula*	He1123	China, Yunnan	JQ716402	–	(He and Li 2013)
	He1157^T^	China, Yunnan	NR_120092	–	(He and Li 2013)
*Hyd.tabacina*	A. M. Ainsworth 2017-1-17 (K(M) 233332)	Great Britain	MK514614	MK890223	(Miettinen et al. 2019)
	Otto Miettinen 22126 (H)	Finland	MK782755	MK787232	(Miettinen et al. 2019)
	Viacheslav Spirin 6066a (H)	Russia, Nizhny Novgorod	MK514600	MK552128	(Miettinen et al. 2019)
*Hyd.tabacinoides*	CLZhao986	China, Yunnan	MG231566	–	Unpublished
	CLZhao859	China, Yunnan	MG231565	–	Unpublished
	Cui10428	China	JQ279604	–	(He and Dai 2012)
***Hyd.yasudai***	**KUC20100409-18**	**Korea**	**PP992265**	**PQ066860**	**This study**
	**KUC20180326-05**	**Korea**	**PP992266**	**PQ066861**	**This study**
	**KUC20210319-14**	**Korea**	**PP992267**	**PQ066862**	**This study**
	**SFC20150707-58**	**Korea**	**PP992268**	**PQ066863**	**This study**
	**SFC20150902-19**	**Korea**	**PP992269**	–	**This study**
	**SFC20160114-04**	**Korea**	**PP992270**	**PQ066864**	**This study**
	**SFC20160512-38**	**Korea**	**PP992271**	–	**This study**
	**SFC20160517-06**	**Korea**	**PP992272**	**PQ066865**	**This study**
	**SFC20160527-41**	**Korea**	**PP992273**	**PQ066866**	**This study**
	**SFC20160614-52**	**Korea**	**PP992274**	–	**This study**
	**SFC20160712-18**	**Korea**	**PP992275**	**PQ066867**	**This study**
	**SFC20180410-24**	**Korea**	**PP992276**	**PQ066868**	**This study**
	**SFC20180712-04**	**Korea**	**PP992277**	**PQ066869**	**This study**
	KUC11055	Korea	KJ713999	–	(Jang et al. 2015)
	KoLRI48661	Korea, Jeju	MT586954	–	Unpublished
	KoLRI_EL005212	Korea, Jeju	MN844835	–	Unpublished
	KoLRI_EL005068	Korea, Jeju	MN844834	–	Unpublished
	**TNS-F78711**	**Japan**	**PP992278**	**PQ066870**	**This study**
	IFO 4969	Japan	AY558598	–	(Jeong et al. 2005)
	Viacheslav Spirin 5533 (H)	Russia, Khabarovsk	MK514597	MK552126	(Miettinen et al. 2019)
	Viacheslav Spirin 6475 (H)	Russia, Primorsky Krai	MK514609	MK552135	(Miettinen et al. 2019)
	CLZhao1495	China, Yunnan	MG231611	–	Unpublished
	CLZhao1475	China, Yunnan	MG231609	–	Unpublished
	CLZhao1486	China, Yunnan	MG231610	–	Unpublished
	CLZhao1422	China, Yunnan	MG231607	–	Unpublished
	He273	China	JQ279614	–	(He and Dai 2012)
	He375	China	JQ279615	–	(He and Dai 2012)
	CLZhao867	China, Yunnan	MG231606	–	Unpublished
	CLZhao933	China, Yunnan	MH114725	–	Unpublished
	CLZhao853	China, Yunnan	MG231605	–	Unpublished
	CLZhao1549	China, Yunnan	MG231612	–	Unpublished
***Hyd.orienticorrugata* sp. nov.**	KUC20121019-16	Korea	KJ668528	–	(Jang et al. 2016)
	**KUC20121123-05**	**Korea**	**PP992279**	–	**This study**
	**KUC20131001-21**	**Korea**	**PP992280**	**PQ066871**	**This study**
	**SFC20140412-06**	**Korea**	**PP992281**	–	**This study**
	**SFC20150212-01**	**Korea**	**PP992282**	**PQ066872**	**This study**
	**SFC20150319-12**	**Korea**	**PP992283**	–	**This study**
	**SFC20150513-06**	**Korea**	**PP992284**	–	**This study**
	**SFC20151030-12^T^**	**Korea**	**PP992285**	**PQ066873**	**This study**
	**SFC20190619-11**	**Korea**	**PP992286**	–	**This study**
	CLZhao938	China, Yunnan	MH114693	–	Unpublished
	He761	China	JQ279606	–	(He and Dai 2012)
	He839	China	JQ279607	–	(He and Dai 2012)
***Hyd.subtabacina* sp. nov.**	**SFC20190322-02^T^**	**Korea**	**PP992287**	**PQ066874**	**This study**
	**SFC20190510-01**	**Korea**	**PP992288**	**PQ066875**	**This study**
	**SFC20190619-15**	**Korea**	**PP992289**	–	**This study**
	Heikki Kotiranta 20797 (H)	Russia, Perm	MK514591	MK552121	(Miettinen et al. 2019)
	Heikki Kotiranta 25205 (H)	Russia, Kransoyarsk	MK514596	MK552125	(Miettinen et al. 2019)
	Otto Miettinen 17028.3 (H)	USA, New York	MK514601	MK552129	(Miettinen et al. 2019)
	Viacheslav Spirin 5196 (H)	Russia, Khabarovsk	MK514602	MK552130	(Miettinen et al. 2019)
	Viacheslav Spirin 6582 (H)	Russia, Khabarovsk	MK514604	–	(Miettinen et al. 2019)
	Viacheslav Spirin 6566 (H)	Russia, Khabarovsk	MK514605	MK552131	(Miettinen et al. 2019)
	Viacheslav Spirin 6520 (H)	Russia, Khabarovsk	MK514607	MK552133	(Miettinen et al. 2019)
	Viacheslav Spirin 6507 (H)	Russia, Khabarovsk	MK514608	MK552134	(Miettinen et al. 2019)
	CFMR:DLL2011-152	USA, Wisconsin	KJ140652	–	(Brazee et al. 2014)
	CFMR:DLL2011-071	USA, Wisconsin	KJ140591	–	(Brazee et al. 2014)
	CFMR:DLL2011-175	USA, Wisconsin	KJ140671	–	(Brazee et al. 2014)
	He810	China	JQ279611	–	(He and Dai 2012)
	He390	China	JQ279610	–	(He and Dai 2012)
*Porodaedaleaalpicola*	Cui12280	China	ON358110	ON631040	(Wu et al. 2019)

^a^The sequences generated in this study are shown in bold. ^T^Indicate the type materials.

Reference ITS and *tef1* sequences for phylogenetic analysis were obtained from GenBank following Miettinen et al. (2019). All sequences were aligned by region using MAFFT v. 7.490 (Katoh and Standley 2013). Sequence alignment and concatenation were performed using Geneious Prime 2023.2.1. (https://www.geneious.com; Suppl. materials 1, 2). A phylogenetic tree was inferred on the CIPRES web portal using the concatenated ITS and *tef1* datasets with maximum likelihood (ML) and Bayesian inference (BI) methods (Miller et al. 2015). For tree inference, ITS and *tef1* sequences were partitioned into eight regions: ITS1, 5.8S, and ITS2 for the ITS region and exon 1, intron 1, exon 2, intron 2, and exon 3 for *tef1* region. jModeltest v. 2.1.10 was used to select the best-fitting substitution model for ITS and *tef1* regions (Darriba et al. 2012). The best-fitting models for ITS1, 5.8S, ITS2, exon 1, intron 1, exon 2, intron 2, and exon 3 regions were HKY+G, JC, HKY+G, K80+G, GTR+I, K80+I, HKY+I, and K80+G, respectively. The ML tree was inferred using RAxML-HPC2 in XSEDE v. 8.2.12 with 1,000 bootstrap replicates (Stamatakis 2014). BI analyses were conducted using MrBayes v. 3.2.3 on XSEDE, with the best model selected for each marker by sampling every 1,000 generations for 20 million generations (Ronquist and Huelsenbeck 2003). The phylogenetic tree was edited using FigTree v. 1.4.3 (Rambaut 2018) and Adobe Illustrator CS6 (Adobe Systems Inc., San Jose, CA, USA).

### ﻿Morphological observation

Macroscopic images of each specimen were captured using a Sony α 6500 camera (Sony, Tokyo, Japan). Microscopic structures were observed using an Olympus BX51 light microscope (Olympus, Tokyo, Japan) at 40–1000 × magnification. The images were captured using a DP20 microscope (Olympus, Tokyo, Japan). At least 20 basidiospores, basidia, and setae were examined to measure the size. Specific color terms were obtained from the Munsell Soil Color Book (Color 2009). The following abbreviations were used: **L** = mean spore length; **W** = mean spore width; **Q** = L/W ratio; **x** = the number of basidiospores measured; **y** = the number of specimens; and **n** = x/y.

## ﻿Results

### ﻿Phylogenetic analyses

**Figure 1.**
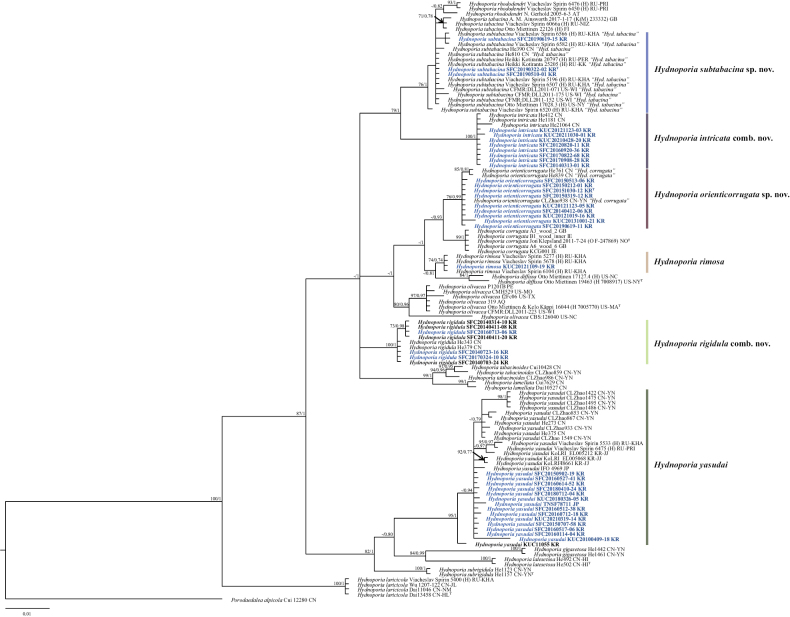
BI tree based on the ITS sequence datasets. The node numbers indicate the bootstrap support values (BS) above 70% and posterior probabilities (PP) over 0.7 as BS/PP. *Hydnoporia* specimens examined in this study are shown in bold. Newly generated sequences in this study are shown in blue and bold. *Porodaedaleaalpicola* (Cui 12280) is used as an outgroup. Letter codes after specimen voucher indicate ISO 3166 country code followed by the origin province. Detailed information is in Table 1. Type specimens are indicated with “T”.

**Figure 2.**
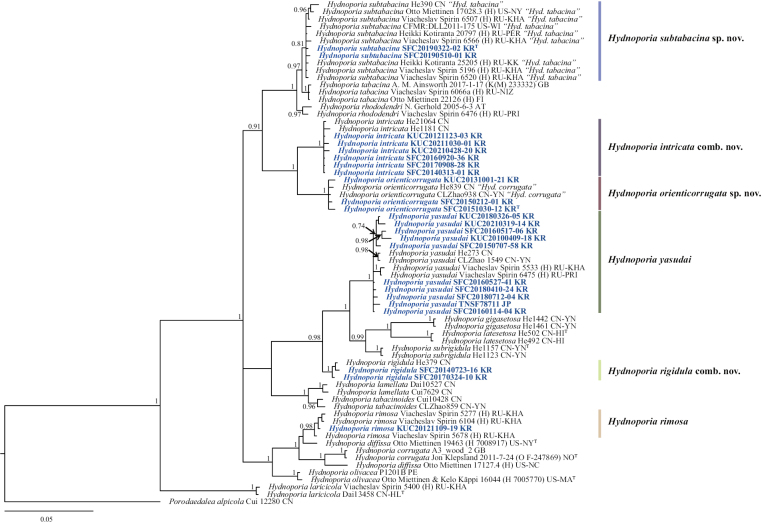
BI tree based on the ITS and *tef1* concatenated sequence datasets. The node numbers indicate the posterior probabilities (PP) above 0.7. *Hydnoporia* specimens examined in this study are shown in bold. Newly generated sequences in this study are shown in blue and bold. *Porodaedaleaalpicola* (Cui 12280) is used as an outgroup. Letter codes after specimen voucher indicate ISO 3166 country code followed by the origin province. Detailed information is in Table 1. Type specimens are indicated with “T”.

Based on ITS sequence analysis, the 43 assessed specimens were phylogenetically grouped within *Hydnoporia* (Fig. 1). Phylogenetic analysis of the ITS and *tef1* regions identified these specimens as six distinct Korean species (Fig. 2, Suppl. material 3). Among them, three were confirmed as previously recorded species in Republic of Korea: *Hymenochaeteintricata* (Lloyd) T. Ito, *Hym.rigidula* Berk. & M.A. Curtis, and *Hydnoporiayasudai* (Imazeki) Spirin & Miettinen. One species was identified as a new record for Korea: *Hyd.rimosa* (Lloyd) Spirin & Miettinen. These four species were also well supported by morphological characteristics.

The remaining two species, previously labelled as “*Hyd.corrugata*” and “*Hyd.tabacina*”, formed distinct clades from their close relatives *Hyd.corrugata* and *Hyd.tabacina*, respectively. However, East Asian and American “*Hyd.tabacina*” were poorly differentiated from European *Hyd.tabacina* and *Hyd.rhododendri* in the ITS phylogeny. Morphological comparison and multimarker-based phylogenetic inference with other *Hydnoporia* species support the recognition of these two as new species, which we propose as *Hyd.orienticorrugata* sp. nov. and *Hyd.subtabacina* sp. nov. Morphological descriptions of the new species are provided in the Taxonomy section.

Additionally, two species previously classified as *Hymenochaete* formed strongly supported clades within *Hydnoporia*. Therefore, we propose their reclassification as *Hyd.intricata* comb. nov. and *Hyd.rigidula* comb. nov.

### ﻿Taxonomy

This section includes morphological description of two new species, *Hyd.orienticorrugata* sp. nov. and *Hyd.subtabacina* sp. nov. and a previously unreported species in Korea, *Hyd.rimosa*.

#### ﻿ Hydnoporia orienticorrugata

Taxon classificationFungiHymenochaetalesHymenochaetaceae

M.Cho, Y.Cho, Y.W.Lim & J.J.Kim sp. nov.

AA854E44-DA96-5F19-8281-60C17BCFC6E9

854671

Fig. 3

##### Diagnosis.

Resupinate, effused basidiome, smooth, brown to reddish brown hymenial surface, sterile margin; cylindrical basidia with 10.3–15.9 × 2.8–4.0 μm, sharp-pointed setae with widened basal part and 35.0–64.6 × 8.1–13.2 μm, narrowly cylindrical to allantoid basidiospores with 4.4–5.8 × 1.5–2.0 μm, and growing on an angiosperm branch (a few on gymnosperm branches).

##### Type.

Korea • Gangwon-do, Pyeongchang-gun, Mt. Heungjeong, 37°65.71'N, 128°32.25'E, alt. 800 m, 30 Oct 2015, Y. W. Lim, (***holotype***: NIBRFG0000516804; ***isotype***: SFC20151030-12).

##### Description.

***Basidiome*** resupinate, effused, thin, covering up to 0.1 mm thick. ***Hymenial surface*** smooth, membranaceous, crustaceous, with many cracks, brown (7.5YR, 4/2) to reddish brown (5YR, 5/3). ***Margin*** sterile, even, concolorous with that of the center. ***Hyphal system*** dimitic; generative hyphae septate, branched, without a clamp connection, thick-walled, few thin-walled, hyaline, 2.7–3.5 μm. Skeletal hyphae aseptate, unbranched, without a clamp connection, thick-walled, reddish-yellow (5YR, 7/8) to yellow (10YR, 7/8), 3.4–4.4 μm.

**Figure 3.**
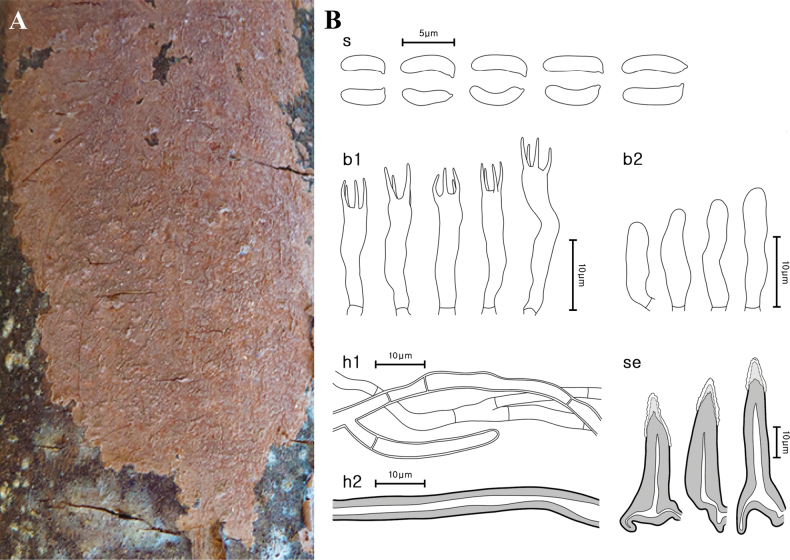
Morphological characteristics of *Hydnoporiaorienticorrugata* (NIBRFG0000516804, holotype) **A** basidiome **B** microscopic features, **s** basidiospores; **b1** basidia; **b2** basidioles; **h1** generative hyphae; **h2** skeletal hyphae; **se** setae. Scale bars: 10 μm (**B**).

***Basidia*** cylindrical, 4-spored, smooth, thin-walled, hyaline, 10.3–15.9(–16.6) × 2.8–4.0 μm. ***Setae*** sharp-pointed, few blunt-pointed, encrusted with crystals, distinctly widened basal part, smooth, bearing narrow lumen, thick-walled, reddish-brown (2.5YR, 4/4), 35.0–64.6 × 8.1–13.2(–14.2) μm. ***Basidiospores*** narrowly cylindrical to allantoid, smooth, slightly curved, thin-walled, hyaline, with narrow apex, a few bearing oil droplets, 4.4–5.8(–6.1) × 1.5–2.0 μm, L = 5.10 μm, W = 1.72 μm, Q = 2.53–3.44, n = 21.

##### Distribution.

East Asia (Korea, China).

##### Ecology.

Grew on an angiosperm branch in mixed hardwood forest, although a few grew on gymnosperm branches.

##### Etymology.

Named after its distribution in East Asian regions and morphological similarity to *Hydnoporiacorrugata*.

##### Additional specimens examined.

Korea • Gangwon-do, Pyeongchang-gun, Odaesan National Park, 37°44.06'N, 128°35.25'E, alt. 690 m, 19 Oct 2012, Y. Jang & S. Jang, KUC20121019-16; Korea • Gangwon-do, Pyeongchang-gun, Odaesan National Park, 37°44.30'N, 128°35.03'E, alt. 660 m, 23 Nov 2012, Y. Jang & S. Jang, KUC20121123-05; Korea • Gangwon-do, Pyeongchang-gun, Odaesan National Park, 37°44.04'N, 128°35.03'E, alt. 680 m, 01 Oct 2013, Y. Jang & S. Jang, KUC20131001-21; Korea • Gangwon-do, Injae-gun, Mt. Bangtae, 37°87.53'N, 128°31.12'E, alt. 390 m, 12 Feb 2015, Y. W. Lim, SFC20150212-01.

##### Notes.

Our specimens were phylogenetically well grouped with the Chinese specimens (He 761, He 839, and CLZhao 938), which were labeled either as *Hymenochaetecorrugata*, *Hymenochaetopsiscorrugata*, or *Pseudochaetecorrugata* (He and Dai 2012; Yang et al. 2016) (Figs 1, 2). Other than these three specimens and KUC20121019-16 from Korea, no other records were found in East Asia, even when the other synonyms of *H.corrugata* were considered. Nevertheless, the East Asian clade formed a distinct clade from the clade with European *Hyd.corrugata*, which included the neotype specimen from Norway (O F-247869). Our findings conform to those of an earlier study, which suggested that sequences identified as *Hyd.corrugata* in Korea and China could represent a novel species (Miettinen et al. 2019). *Hydnoporiaorienticorrugata* sp. nov. has micromorphological characteristics similar to those of *Hyd.corrugata*, but the latter has a grey to pale brown hymenial surface (Miettinen et al. 2019) that differs from the new species. Additionally, *Hyd.orienticorrugata* occurs on angiosperm and gymnosperm branches in Korea and China (He and Dai 2012) whereas *H.corrugata* occurs only on angiosperm branches and seems to be restricted to Europe (Austria, England, Ireland, Norway, Russia, and Sweden) (Fries 1815; Grundy et al. 2012; Miettinen et al. 2019).

#### ﻿ Hydnoporia subtabacina

Taxon classificationFungiHymenochaetalesHymenochaetaceae

M.Cho, Y.Cho, Y.W.Lim & J.J.Kim sp. nov.

3512CF47-456B-5697-BB5F-FA39827A090F

854672

Fig. 4

##### Diagnosis.

Effused-reflexed, pileate basidiome, smooth, brown hymenial surface, sterile margin; cylindrical basidia with 14.6–17.9 × 2.9–3.8 μm, sharp-pointed and few elongated setae with 58.6–140.0 × 9.8–26.1 μm, narrowly cylindrical basidiospores with 4.4–5.7 × 1.6–1.9 μm, and occurs on angiosperm trees (branches and trunks).

##### Type.

Korea • Gyeongsangbuk-do, Bonghwa-gun, Taebaeksan National Park, Baekcheon valley, 37°00.64'N, 128°98.41'E, alt. 830 m, 22 Mar 2019, Y. W. Lim & S. Yoo, (***holotype***: NIBRFG0000505378; ***isotype***: SFC20190322-02).

##### Description.

***Basidiome*** effused-reflexed, pileate, 0.1 mm thick. ***Hymenial surface*** smooth, membranaceous, brown (7.5YR, 5/4) to dark brown (7.5YR, 3/3). ***Margin*** sterile, slightly lighter. ***Hyphal system*** dimitic; generative hyphae septate, frequently branched, without a clamp connection, thick-walled, hyaline, 2.3–3.8 μm. Skeletal hyphae aseptate, unbranched, without a clamp connection, thick-walled, reddish-yellow (5YR, 7/8) to yellow (10YR, 7/8), 3.0–5.0 μm.

**Figure 4.**
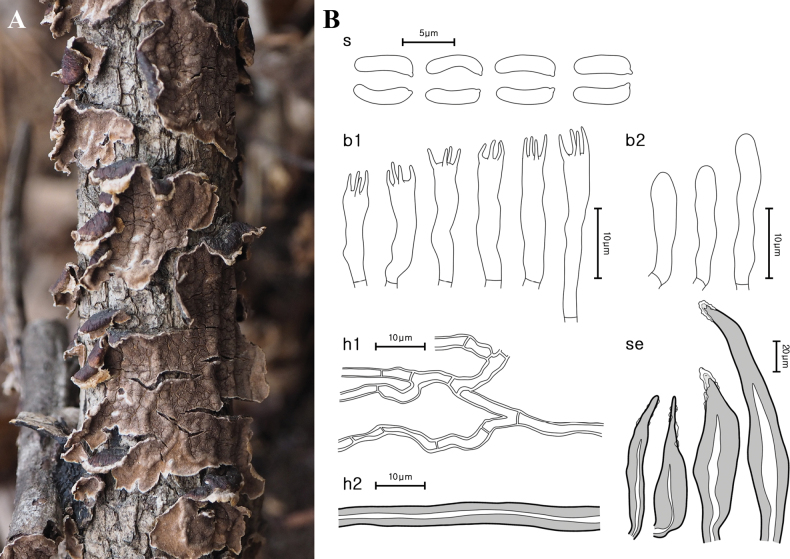
Morphological characteristics of *Hydnoporiasubtabacina* (NIBRFG0000505378, **holotype**) **A** basidiome **B** microscopic features, **s** basidiospores; **b1** basidia; **b2** basidioles; **h1** generative hyphae; **h2** skeletal hyphae; **se** setae. Scale bars: 10 μm (**s**, **b1**, **b2**, **h1**, **h2**); 20 μm (**se**).

***Basidia*** cylindrical, 4-spored, smooth, thin-walled, hyaline, (14.0–)14.6–17.9(–18.7) × 2.9–3.8 μm. ***Setae*** sharp-pointed, encrusted with crystals, cylindrical, fusiform, few elongated apex, smooth, bearing a narrow or wide lumen, thick-walled, dark reddish brown (5YR, 3/4) to dusky red (2.5YR, 3/2), 58.6–140.0 × 9.8–26.1 μm. ***Basidiospores*** narrowly cylindrical, smooth, slightly curved, thin-walled, hyaline, with narrow apex, 4.4–5.7 × 1.6–1.9 μm, L = 5.22 μm, W = 1.68 μm, Q = 2.78–3.61, n = 21.

##### Distribution.

Korea, China, Far East Russia, and US.

##### Ecology.

Grew on *Quercus* in angiosperm forest.

##### Etymology.

Named after its morphological similarity with *Hydnoporiatabacina*.

##### Additional specimens examined.

Korea • Gangwon-do, Taebaek-si, Taebaeksan National Park, Yuilsa Temple, 37°10.87'N, 128°91.07'E, alt. 1,250 m, 10 May 2019, Y. W. Lim & S. Yoo, SFC20190510-01.

##### Notes.

According to our phylogenetic analysis, sequences annotated as ‘*Hyd.tabacina*’ were divided into a European and an Asian-North American clade (Fig. 2). The holotype specimen sequence is unavailable, but it is known that the specimen (≡*Ariculariatabacina* Sowerby) locality is Britain (Sowerby 1797) and the lectotype specimen is from Sweden (Miettinen et al. 2019). Therefore, we acknowledge the European clade (Finland, Great Britain, and Western Russia) as *Hyd.tabacina* and the Asian-American clade (China, Fareast Russia, Korea, and the US) as the new species, following the results of a previous study (Miettinen et al. 2019). *Hydnoporiasubtabacina* sp. nov. occurs only on angiosperm branches or trunks. The microscopic characteristics of *Hyd.subtabacina* and *Hyd.tabacina* are similar, but longer and wider basidiospores are reported in the latter species, viz. 4.58–5.9 × 1.78–2.02 μm (Miettinen et al. 2019). Further, setal measurements of our specimens had broader variation (58.6–140.0 × 9.8–26.1 μm) compared to those of the Fareast Russian (63.92–94.15 × 9.38–14.5 μm) and North American specimens (71.1–97.9 × 9.5–14.23 μm) (Miettinen et al. 2019). While there is a morphological description of Chinese ‘*Hyd.tabacina*’, no sequence data were available for these observed specimens (Dai 2010). Additionally, the morphological characteristics of the Chinese ‘*Hyd.tabacina*’ with a hydnoid and yellowish basidiome (Dai 2010) differ from those of the European specimens and of *Hyd.subtabacina*. Therefore, further research is needed for an accurate identification of Chinese ‘*Hyd.tabacina*’.

#### ﻿ Hydnoporia rimosa

Taxon classificationFungiHymenochaetalesHymenochaetaceae

(Lloyd) Spirin & Miettinen, Fungal Systematics and Evolution 4: 92 (2019)

9AADF867-B21D-5C6F-A1A2-6EA0261408F4

830597

Fig. 5

##### Diagnosis.

Resupinate, effused basidiome, reddish brown hymenial surface, white to brown margin; cylindrical to narrowly clavate basidia with 10.0–14.1 × 2.7–3.4 μm, sharp to blunt pointed setae with 44.4–83.1 × 8.2–13.4 μm, narrowly cylindrical to allantoid basidiospores with 4.7–6.1 × 1.7–2.0 μm, and occurs on angiosperm branches.

##### Type.

Japan • Tohoku, Sendai, 24 Oct 1920, Yasuda, (***holotype***: TNS-F203210; ***lectotype***: MBT387146).

##### Description.

***Basidiome*** resupinate, effused, thin, leathery, up to 0.2 mm thick. ***Hymenial surface*** smooth, membranaceous, crustaceous, with many cracks, light reddish-brown (2.5YR, 6/4) to reddish-brown (2.5YR, 5/3). ***Margin*** sterile, even, edge whitish (7.5YR, 9/2) when fresh and becomes brown (7.5YR, 5/8). ***Hyphal system*** dimitic; generative hyphae septate, branched, without a clamp connection, thick-walled, hyaline to pale brown (10YR, 6/3), 3.0–3.5 μm. Skeletal hyphae aseptate, unbranched, without a clamp connection, thick-walled, yellowish (7.5YR, 6/8) to reddish brown (2.5YR, 4/3), 3.5–4.2 μm.

***Basidia*** cylindrical to narrowly clavate, 4-spored, smooth, thin-walled, hyaline, 10.0–14.1(–14.5) × 2.7–3.4 μm. ***Setae*** sharp- to blunt-pointed, encrusted with crystals, smooth, bearing a wide or narrow lumen, thick-walled, projecting up to 15 µm above the hymenium, dark reddish brown (2.5YR, 2.5/3), 44.4–83.1 × 8.2–13.4(–18.6) μm. ***Basidiospores*** narrowly cylindrical to allantoid, smooth, thin-walled, hyaline, with narrow apex, 4.7–6.1 × (1.5–)1.7–2.0 μm, L = 5.40 μm, W = 1.80 μm, Q = 2.64–3.30, n = 20.

##### Specimen examined.

Korea • Gangwon-do, Pyeongchang-gun, Odaesan National Park, 37°44.30'N, 128°35.01'E, alt. 660 m, 9 Nov 2012, Y. Jang & S. Jang, KUC20121109-19.

##### Notes.

The observed *Hyd.rimosa* specimen from Korea has similar morphological characteristics as those of the type specimen (TNS-F203210), but the type specimen has abundant blunt-pointed setae and wider basidiospores (L = 5.22, W = 1.99, Q = 2.47–2.78) (Miettinen et al. 2019) compared to the Korean specimen. In the phylogenetic tree, *Hyd.diffissa* is closely related to *Hyd.rimosa*. These two species are practically indistinguishable in morphology, except that *Hyd.rimosa* has a wider lumen and greater size variation of setae (Miettinen et al. 2019). However, they are geographically distinct, where *Hyd.diffissa* is distributed across North and South America (Colombia, Peru, and Eastern US) (Miettinen et al. 2019), while *Hyd.rimosa* is distributed in East Asia (Japan, Korea, and Far East Russia) (Ito 1930b; Miettinen et al. 2019).

**Figure 5.**
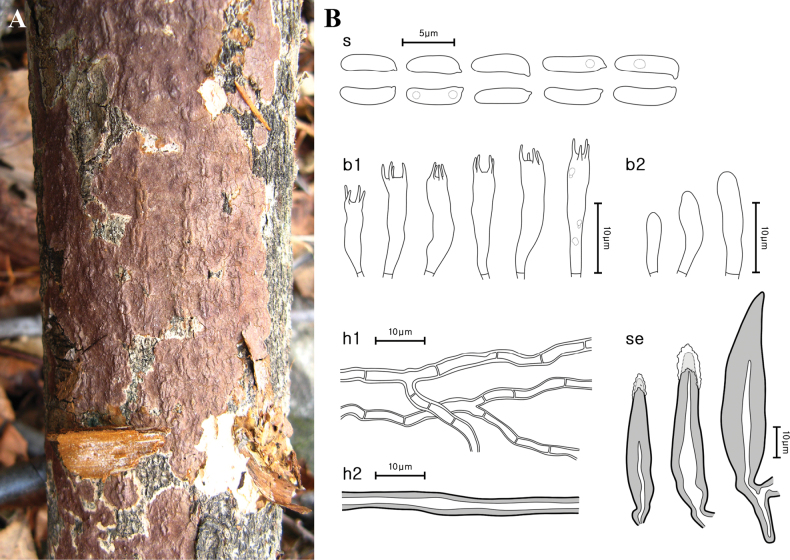
Morphological characteristics of *Hydnoporiarimosa* (KUC20121109-19) **A** basidiome **B** microscopic features, **s** basidiospores; **b1** basidia; **b2** basidioles; **h1** generative hyphae; **h2** skeletal hyphae; **se** setae. Scale bars: 10 μm (**B**).

#### ﻿ Hydnoporia intricata

Taxon classificationFungiHymenochaetalesHymenochaetaceae

(Lloyd) M.Cho, Y.Cho, Y.W.Lim & J.J.Kim comb. nov.

852C2243-D6CB-50C7-8E55-EFBE8D73136E

854673

Stereum intricatum Lloyd, Mycol. Writ. 7(67): 1157, 1922. Basionym.
Hymenochaete intricata (Lloyd) T.Ito, in Tokyo, Bot. Mag. 44: 156, 1930. Synonyms.
=Pseudochaeteintricata (Lloyd) S.H.He & Y.C.Dai, Fungal Diversity, 56: 89, 2012.
=Hymenochaetopsisintricata (Lloyd) S.H.He & Jiao Yang, Mycol. Prog. 15(2/13): 6, 2016.

#### ﻿ Hydnoporia rigidula

Taxon classificationFungiHymenochaetalesHymenochaetaceae

(Berk. & M.A.Curtis) M. Cho, Y.Cho, Y.W.Lim & J.J.Kim comb. nov.

1986A0B2-3CD7-5433-A143-1DF9A122036B

854674

Hymenochaete rigidula Berk. & M.A.Curtis, Journal of the Linnean Society. Botany 10: 334, 1869. Basionym.
Pseudochaete rigidula (Berk. & M.A.Curtis) S.H.He & Y.C.Dai, Fungal Diversity 56: 89, 2012. Synonyms.
=Hymenochaetopsisrigidula (Berk. & M.A.Curtis) S.H.He & Jiao Yang, Mycol. Prog. 15 (2/13): 6, 2016.

### ﻿Taxonomic key to *Hydnoporia* in Korea

**Table:** 

1	Basidiome resupinate	**2**
–	Basidiome effused-reflexed	**5**
2	Basidiome margin strictly attached to substrate	**3**
–	Basidiome margin detached from substrate	**4**
3	Setae sharp-pointed, distinctly widened base	***Hyd.orienticorrugata***
–	Setae sharp– to blunt-pointed, narrow base	***Hyd.rimosa***
4	Occurs on dead angiosperm branches, setae 29.1–66.4 × 5.0–9.2 μm	***Hyd.rigidula***
–	Occurs on dead gymnosperm branches, setae 36.0–92.7 × 9.1–19.6 μm	***Hyd.yasudai***
5	Basidiospores cylindrical, Q value < 3.0	***Hyd.subtabacina***
–	Basidiospores allantoid, Q value > 3.0	***Hyd.intricata***

## ﻿Discussion

Some *Hydnoporia* species are indistinguishable based on a phylogenetic tree that is inferred from ITS data alone, notably as *Hyd.rhododendri*, *Hyd.tabacina*, and *Hyd.subtabacina* (Fig. 1). This is resolved by including an additional genetic marker, *tef1*, to infer a multigenetic marker phylogeny (Fig. 2). A phylogenetic analysis solely based on ITS for fungal species identification has been criticized by taxonomists (Harder et al. 2013; Santos et al. 2017). The use of ITS alone works well for several genera (Frøslev et al. 2007; Hallenberg et al. 2007). However, it may lead to under-splitting of some taxa (Harder et al. 2013) or over-splitting of other, taxa as seen for *Hyd.yasudai* (Fig. 1). Therefore, the use of additional protein-coding genetic markers for phylogenetic analyses is essential to achieve properly resolved species clades (Fig. 2, Suppl. material 1).

To the 14 previously accepted species in *Hydnoporia*, the present study adds four ones: two new *Hydnoporia* species were described, and two other species were transferred to *Hydnoporia*. Of these 18 species, 13 have been reported in East Asia (China, Japan, Korea, and Fareast Russia), namely *Hydnoporiagigasetosa*, *Hyd.intricata*, *Hyd.lamellata*, *Hyd.laricicola*, *Hyd.latesetosa*, *Hyd.orienticorrugata* sp. nov., *Hyd.rhododendri*, *Hyd.rigidula*, *Hyd.rimosa*, *Hyd.subrigidula*, *Hyd.subtabacina* sp. nov., *Hyd.tabacinoides*, and *Hyd.yasudai* (Miettinen et al. 2019). Regarding the remaining five species, *Hyd.diffissa*, *Hyd.lenta*, and *Hyd.olivacea* have only been reported in the Americas, and *Hyd.corrugata* and *Hyd.tabacina* have only been reported in Europe (Miettinen et al. 2019). This indicates that many *Hydnoporia* species are geographically or ecologically restricted unlike many other wood-decaying fungi that are cosmopolitan and less constrained by environmental factors (Sato et al. 2012). The regional constraint for *Hydnoporia* may be the consequence of host or vector specificity.

In Korea, three previously recorded species – namely *Hydnoporiaintricata*, *Hyd.rigidula*, and *Hyd.yasudai* – have been phylogenetically verified. Clades of the first two species were supported by high bootstrap values and posterior probabilities. However, *Hyd.yasudai* formed a complex, as observed by Miettinen et al. (2019), who suggested dividing the *Hyd.yasudai* complex into three to six different species. In our study, *Hyd.yasudai* specimens had large sequence variations in ITS (13 bp, 1.3%) and *tef1* (20 bp, 3.4%) but morphologically, the spore sizes were relatively constant among the Fareast Russian, Japanese, and Korean specimens (Miettinen et al. 2019). Additionally, *Hyd.yasudai* has a specific host preference for gymnosperms (*Pinus* spp., *Abiesfirma*, and *A.holophylla*, etc.) (Léger 1998; Dai 2010; Miettinen et al. 2019). Therefore, we proposed the *Hyd.yasudai* complex to remain as a single species, with variation based on geographical distribution. Two *Hydnoporia* species previously reported in Korea, *Hyd.corrugata* and *Hyd.tabacina*, were each represent other species. East Asian ‘*Hyd.corrugata*’ specimens (Korean and Chinese) (He and Dai 2012) were phylogenetically separated from the European *Hyd.corrugata*, which contains the type specimen (O F-247869) (Figs 1, 2), and it was thus introduced as a new species, *Hyd.orienticorrugata* sp. nov. This result was consistent with that of Miettinen et al. (2019). Similarly, East Asian and North American ‘*Hyd.tabacina*’ were different from the holotype – a European specimen – both phylogenetically and morphologically (Figs 2, 4), and they were introduced as a new species, *Hyd.subtabacina* sp. nov.

Two species combinations are proposed, viz. *Hyd.intricata* comb. nov. and *Hyd.rigidula* comb. nov. *Hydnoporiaintricata* was first described as *Stereumintricatum* by Lloyd in 1922. It was then renamed to *Hymenochaeteintricata* (Ito 1930a). After decades, He & Dai renamed it to *Pseudochaeteintricata* (He and Dai 2012) and then Yang suggested *Hymenochaetopsisintricata* (Yang et al. 2016). Based on a recent study, *Pseudochaete* and *Hymenochaetopsis* are no longer valid and are considered younger synonyms of *Hydnoporia* (Miettinen et al. 2019). Based on this study, *Hymenochaeteintricata* is verified to belong to *Hydnoporia.* The morphological characteristics of the Korean *Hyd.intricata* specimens studied here correspond to those of the original description. Therefore, we suggest that *Hymenochaeteintricata* should be included in *Hydnoporia*. This result was further supported by phylogenetic analysis of combined sequence datasets (ITS+*tef1*) with high bootstrap support value and posterior probability (Fig. 2, Suppl. material 1).

*Hydnoporiarigidula* was initially reported as *Hymenochaeterigidula* Berk. & M. A. Curtis in 1868 (Berkeley and Curtis 1868). Based on multiple taxonomic revisions, it was renamed *Pseudochaeterigidula* (He and Dai 2012) and later *Hymenochaetopsisrigidula* according to (Yang et al. 2016). According to Miettinen et al. (2019) and this study, *Hymenochaeterigidula* is phylogenetically located in *Hydnoporia*. Miettinen et al. (2019) also stated that East Asian *Hyd.rigidula* may be distinct from the American *Hyd.rigidula*. However, no sequenced specimens are available from the American or Caribbean (Cuba and Jamaica) regions (He and Dai 2012; Yang et al. 2016) to verify the differences. Nevertheless, East Asian and American specimens have similar setae and basidiospore size measurements and other macro-morphological characteristics (Parmasto 2001; He and Dai 2012; Kim et al. 2016). Therefore, further assessment is required to separate the species.

In conclusion, we propose two new species and two species combinations within the genus *Hydnoporia*. Given the morphological similarities between *Hydnoporia* and *Hymenochaete*, molecular analysis is crucial for accurate species identification, ideally using multiple genetic regions. This study resolved the taxonomic confusion arising from the continuous systematic revision of some *Hydnoporia* species and emphasized the need to update old names to avoid confusion. Although *Hydnoporia* appears to be primarily distributed in East Asia, with a few species in Europe and the Americas, it remains underexplored in the Southern Hemisphere. Therefore, further investigation of the global distribution and biogeographical relationships of *Hydnoporia* is necessary to understand the true diversity of the genus and establish a stable species classification.

## Supplementary Material

XML Treatment for Hydnoporia orienticorrugata

XML Treatment for Hydnoporia subtabacina

XML Treatment for Hydnoporia rimosa

XML Treatment for Hydnoporia intricata

XML Treatment for Hydnoporia rigidula

## ﻿Additional information

### Author ORCIDs

Minseo Cho https://orcid.org/0009-0007-0834-7294

Yoonhee Cho https://orcid.org/0000-0002-0743-0914

Sun Lul Kwon https://orcid.org/0000-0002-2075-5342

Dohye Kim https://orcid.org/0009-0007-0551-099X

Kentaro Hosaka https://orcid.org/0000-0002-4469-8303

Young Woon Lim https://orcid.org/0000-0003-2864-3449

Jae-Jin Kim https://orcid.org/0000-0001-8990-2139

### Data availability

All of the data that support the findings of this study are available in the main text or Supplementary Information.

## Supplementary materials

Supplementary material 1 Data 1 Data type fasta Explanation note ITS sequence alignments of all studied sequence datasets. https://binary.pensoft.net/file/1182972This dataset is made available under the Open Database License (http://opendatacommons.org/licenses/odbl/1.0/). The Open Database License (ODbL) is a license agreement intended to allow users to freely share, modify, and use this Dataset while maintaining this same freedom for others, provided that the original source and author(s) are credited.Minseo Cho, Yoonhee Cho, Sun Lul Kwon, Dohye Kim, Kentaro Hosaka, Young Woon Lim, Jae-Jin Kim

Supplementary material 2 Data 2 Data type fasta Explanation note Concatenated multimarker (ITS+*tef1*) sequence alignments of studied sequence datasets. https://binary.pensoft.net/file/1182973This dataset is made available under the Open Database License (http://opendatacommons.org/licenses/odbl/1.0/). The Open Database License (ODbL) is a license agreement intended to allow users to freely share, modify, and use this Dataset while maintaining this same freedom for others, provided that the original source and author(s) are credited.Minseo Cho, Yoonhee Cho, Sun Lul Kwon, Dohye Kim, Kentaro Hosaka, Young Woon Lim, Jae-Jin Kim

Supplementary material 3 ML tree based on the ITS and *tef1* concatenated sequence datasets Data type png Explanation note The node numbers indicate the bootstrap support values (BS) above 70%. *Hydnoporia* specimens examined in this study are shown in bold. Newly generated sequences in this study are shown in blue and bold. *Porodaedaleaalpicola* (Cui 12280) is used as an outgroup. Letter codes after specimen voucher indicate ISO 3166 country code followed by the province of origin. Type specimens are indicated with “T”. https://binary.pensoft.net/file/1183075This dataset is made available under the Open Database License (http://opendatacommons.org/licenses/odbl/1.0/). The Open Database License (ODbL) is a license agreement intended to allow users to freely share, modify, and use this Dataset while maintaining this same freedom for others, provided that the original source and author(s) are credited.Minseo Cho, Yoonhee Cho, Sun Lul Kwon, Dohye Kim, Kentaro Hosaka, Young Woon Lim, Jae-Jin Kim
