# Supplementary figures and images for: ﻿Taxonomic study of Hydnoporia (Hymenochaetales, Hymenochaetaceae) in East Asia with two new species
Source: MycoKeys. 2024 Nov 25;111:1–20. doi: 10.3897/mycokeys.111.137347 (PMC11612637; doi:10.3897/mycokeys.111.137347)

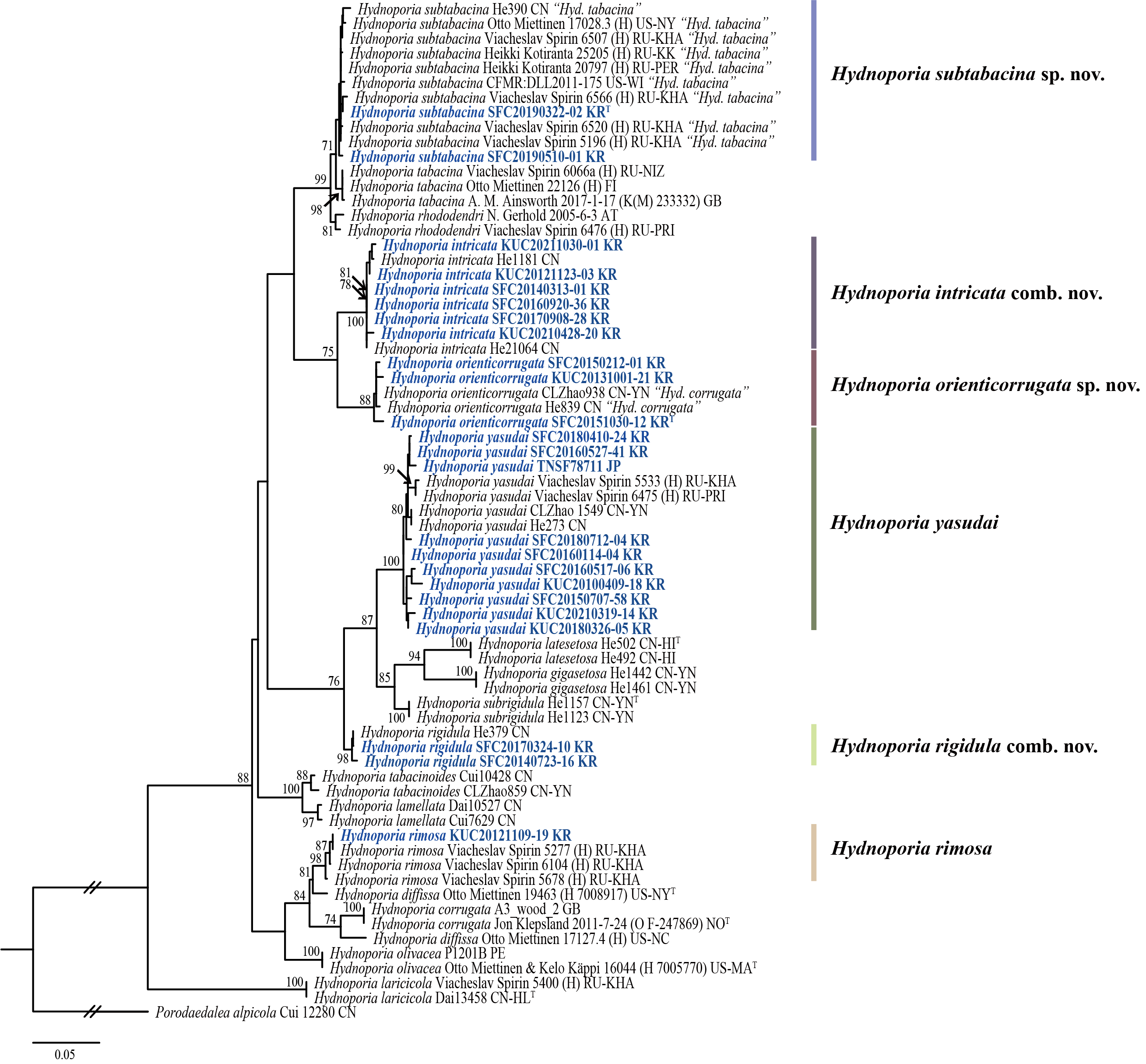

Supplement: Supplementary material 3 — ML tree based on the ITS and tef1 concatenated sequence datasets [file mycokeys-111-001-s003.png]
